# “On an island by myself”: implications for the inclusion of autistic students in self-contained classrooms in public elementary schools

**DOI:** 10.3389/fpsyt.2023.1241892

**Published:** 2023-09-26

**Authors:** Kaitlyn Ahlers, Maria L. Hugh, Daina Tagavi, Curtis Eayrs, Alyssa M. Hernandez, Theodore Ho, Jill Locke

**Affiliations:** ^1^Department of Psychiatry, Geisel School of Medicine, Dartmouth College, Lebanon, NH, United States; ^2^Department of Psychiatry, Dartmouth Hitchcock Medical Center, Lebanon, NH, United States; ^3^Department of Special Education, School of Education, University of Kansas, Lawrence, KS, United States; ^4^Department of Psychology, College of Arts and Sciences, University of Washington, Seattle, WA, United States; ^5^Department of Psychiatry and Behavioral Sciences, School of Medicine, University of Washington, Seattle, WA, United States; ^6^Department of Psychology, School of Arts and Sciences, University of Pennsylvania, Philadelphia, PA, United States; ^7^Seattle Children's Research Institute, Seattle, WA, United States

**Keywords:** autism, inclusion, special education, elementary school, educators

## Abstract

**Introduction:**

Autistic students have limited access to inclusive classes and activities in their schools. Principals and special education teachers who directly teach and administer programs for autistic elementary students can offer critical insight into factors, such as educators’ attitudes, that may impact inclusive opportunities in schools. These attitudes may serve as barriers to or facilitators of promoting an inclusive school setting.

**Methods:**

Semi-structured interviews with 26 elementary school principals and 26 special education teachers explored their experiences implementing evidence-based practices for autistic students (pivotal response training, discrete trial training, and visual schedules) in 26 self-contained classrooms in the United States. Autism-specific culture and inclusion emerged as a theme, which was analyzed for this paper.

**Results:**

An inductive approach to thematic analysis revealed principals’ and special education teachers’ perspectives regarding the “autism-specific culture” in the school, including attitudes towards and inclusion of autistic students in self-contained classrooms in the broader school environment. Analysis of text related to “autism-specific culture” detailed aspects of inclusion, factors (i.e., barriers and facilitators) affecting inclusion, principals’ and special education teachers’ attitudes towards autistic students placed in self-contained classrooms, attitudes of other school staff towards teachers in self-contained classrooms, and recommendations to support an inclusive school environment for autistic students.

**Discussion:**

Results suggest that valuing “equal” access to classes and activities for autistic students in self-contained classrooms may not be sufficient for promoting an inclusive school environment, Educators may benefit from targeted strategies to facilitate inclusion. Strategies range from supporting educators’ attitudes and knowledge of autism to shifting physical aspects of the school environment (e.g., location of classrooms). Additional implications for supporting the true inclusion (i.e., inclusion that goes beyond physical inclusion) involves of autistic students in self-contained classrooms schools are discussed.

## Introduction

In the United States, the prevalence of autism spectrum disorder (ASD) has increased to about one in 36 children (1). With that, there is an increase in the rates of autistic students being served in public schools (2), the primary service setting for autistic youth (3). It is imperative to include autistic students in general education settings alongside typically developing peers (4–6). Nevertheless, across the United States, only approximately 30% of autistic students were served up to 80% of their day in general education settings (7). Self-contained classrooms (i.e., those that only serve students with disabilities) persist as a common placement (8). While placing students in the same location as peers is a necessary start to dismantle segregated placements and practices, placement alone is insufficient to realize the educational and social benefits of inclusion (9). Autistic students may be excluded from inclusive contexts associated with many factors, including low knowledge, negative attitudes, or stigma by school professionals or peers (10). Ideally, all aspects of the school are designed to support inclusion through its structure, norms, practices, and culture, to create the context for all students to participate in their classrooms and have a sense of belonging to their school communities (11–13). To improve inclusion rates and practices, educators’ experiences and perspectives on stigmatization of autism and what can facilitate effective inclusion are needed.

### A variety of educational placements

Students with disabilities should be taught with their non-disabled peers to the greatest extent possible and receive specialized intervention support that meets their needs (4). Although this has been written into law since 1975 (4), the predominant approach for placement of students has been exclusion, where students with disabilities are served in separate classrooms from their neurotypical peers. Educators often cite that the specialized service needs of students with disabilities [e.g., speech-language intervention, occupational therapies, behavioral therapy (14)] are challenging to integrate into general education settings (15, 16), and researchers have demonstrated that social stigma toward autistic students and those with other disabilities can impede inclusion (17, 18). The amount of time children with disabilities spend with their non-disabled peers placed in general education settings rests on a continuum of educational placements. On the two ends of this continuum are self-contained classrooms, where only students with disabilities are members, and general education settings, which predominantly include students without disabilities.

Self-contained settings usually have a lower student-teacher ratio and use personalized goals and curricula for students based on their needs; often, students may vary in grade level or age within this setting (19). Students’ goals can cover various developmental domains (e.g., adaptive, social communication, physical, and cognitive) that influence their academic achievement. The rationale for serving students in these settings is that children may have more teacher attention and fewer distractions, though this often is not the case (19). Moreover, IEP quality has not been demonstrated to improve in quality by placement [i.e., self-contained vs. inclusive; (20)]. In contrast, general education settings focus on a general curriculum and standards that all students are expected to meet. Although general education classrooms include tailored support for students within multi-tiered systems of support, [MTSS (21)] a system of supports that provides specific practices based on students’ level of need, standard educational perspectives are that there is less room for variation in the focal skill areas within general education classrooms (e.g., primarily academics with some social–emotional focus). There is a tension, however, that all students with disabilities should access the general curriculum and their neurotypical peers, which requires schools to offer alternative models.

To meet the expectations of supporting students with disabilities in their least restrictive environment, accessing the general curriculum, and balancing their support needs, U.S. public schools offer different models of inclusion that primarily relate to time spent in a general education classroom. These include: a) hybrid, which is some time in self-contained and some time in general education, b) push-in, which includes time in general education with special education service support, c) pull-out, which is time in a general education classroom and then the student receives special education service support in a separate setting, or d) inclusive classrooms, where students’ individualized education program (IEP) services and goals are addressed in the general education classroom integrated into the classroom activities with their non-disabled peers (7).

Given the mandates of IDEA and the recognized benefits of inclusion, educators have increasingly sought alternative models to self-contained classrooms, intending to increase the access of students with disabilities to the general education setting and non-disabled peers. In an inclusive classroom model, the general classroom is set up with all students with and without disabilities in mind to provide both class-wide and individualized supports (22), which helps support autistic students’ rightful presence in all spaces and meet legal expectations. Our co-author CE, an ASD advocate, defines inclusion as: “the ongoing process to remove institutional and structural barriers that have been in place for many years that prevent a more equitable educational outcome for ASD students. An important element of this definition of inclusion is that ASD students have a ‘seat at the table’ with effective parent/guardian advocacy for general education inclusion classes on their behalf.” This definition aligns with some presented in the literature of equity-based inclusion, meaning all children receive the levels and types of support and instruction they need (22) and recognizes that barriers to attaining this level of inclusion remain in the school systems.

### School staff and factors supporting inclusion

Moving the needle on inclusion in a way that aligns with this definition requires school staff to work across levels [e.g., student-level, teacher-level, school-level, district-level (23)]. Federal and state-level policies set the context and requirements for inclusion, but individual schools and classroom leaders create the conditions for inclusion. Within a school, the staff comprises general education teachers, special education teachers, related service providers (e.g., occupational therapists, speech-language pathologists), principals, and other administrators. To facilitate effective inclusion for autistic students, school staff need adequate resources, support, and collaboration across levels (24). Principals also play an essential role in providing the necessary implementation leadership [i.e., support of adopting new practices; (22, 25)] as well as make structural decisions (e.g., classroom space assignments, staff allocation, caseloads) to enact inclusion. Importantly, teachers identify that some of their primary strategies for including autistic students are advocacy within their school systems for training and resources and collaboration with other educators (26).

Malleable educator-level factors, such as their attitudes and stigma towards autism and inclusion, are likely instrumental in supporting autistic students’ inclusive service delivery in general education classes. Educators describe that the inclusion climate and culture across their schools require disability awareness and education, often grounded in educators’ positive attitudes (26). School staff’s attitudes toward autism and the inclusion of autistic students is frequently identified as a barrier to inclusion (26) and influential to effective practices in inclusive contexts for autistic students (27). Similarly, for other groups of students with disabilities, such as attention-deficit/hyperactivity disorder (ADHD), educators’ attitudes towards inclusion have been influenced by stigma associated with perceptions of the condition or difference [i.e., ADHD; (28)]. Thus, stigma toward autism and the inclusion of autistic students with non-disabled peers may continue to influence educators’ attitudes and impede inclusion and student participation across all social contexts (28, 29). Importantly, principals’ and teachers’ attitudes also facilitate inclusion when they are accepting, favorable toward autism, and understanding of students’ individual differences (30, 31). Therefore, school staff’s individual attitudes and a collective positive culture toward autism and inclusion may be key to improving autistic students’ access to inclusive classrooms.

### Study purpose

In the last decade, the proportion of students with disabilities accessing general education classrooms has remained somewhat stagnant (32). The persistent need to support inclusion presents opportunities to learn from those who play key roles in supporting inclusive placements of autistic students [i.e., principals and special education teachers (31)]. Special education teachers who work primarily with autistic students in self-contained settings offer unique autism expertise given their daily classroom experiences and involvement in special education teams where placement decisions are made. As part of a larger mixed-methods study (33) aimed at understanding contextual factors that influence special education teachers’ fidelity to implementing autism-focused evidence-based practices (EBPs), *autism-specific culture and inclusion* arose inductively. *Autism-specific culture and inclusion* refers to the attitudes, perspectives, and treatment of autistic students and staff who support them in self-contained classrooms and inclusion (or lack thereof) in general education classrooms and other school spaces (e.g., lunchroom) with non-disabled peers. In response to this, this qualitative study aimed to characterize how principals and special education teachers perceive the “autism culture” in their schools, as it relates to their and others’ perspectives of autism and inclusive practices for autistic students in self-contained settings in public elementary schools. Thus, this paper describes principals’ and special education teachers’ perspectives regarding the “autism culture” in their schools and its implications for the inclusion of autistic students in schools with traditionally segregated autism-specific settings.

## Materials and methods

### Participants and setting

Data were drawn from a larger study that examines how contextual factors influence special education teachers’ fidelity to three EBPs [i.e., discrete trial training, pivotal response training, and visual schedules (34–37)] for autistic youth (27, 33, 38). In brief, 26 schools with kindergarten through third-grade special education classrooms located in the northeastern United States were included in this study. Enrolled schools received training in three autism-focused EBPs based on the principles of applied behavioral analysis. At the start of the school year, teachers received training in the three EBPs, followed by monthly coaching in each of those EBPs. From January to April of the academic year in which data were collected, fidelity observations were conducted in special education classrooms. Teachers were then purposefully sampled based on their average levels of fidelity (i.e., high vs. low) across the three EBPs to participate in qualitative interviews during April and May of the same year. This paper reports on the qualitative interviews, and reporting is guided by the Consolidated Criteria for Reporting Qualitative Research (COREQ) guidelines [(39); see Supplementary File S1].

To capture a full range of teacher experiences related to individual- and school-level factors associated with EBP implementation, special education teachers with high (i.e., in the top tertile based on their average fidelity rating across EBPs) and low (i.e., in the bottom tertile across EBPs) fidelity were invited to complete interviews. Principals of each special education teacher also were invited to participate in interviews. Potential participants were invited via email. Interviews with participants from thirteen high-fidelity and thirteen low-fidelity classrooms were sufficient to achieve data saturation (40).

Participants included *n* = 26 principals and *n* = 26 special education teachers who completed qualitative interviews. Educator characteristics are presented in Table 1. Both principals (77%) and special education teachers (92%) were predominately female. Principals were racially and ethnically diverse with representation across Asian (*n* = 1), Black (*n* = 12), white non-Hispanic (*n* = 10), and Latine groups (*n* = 4); in contrast, special education teachers were predominantly white non-Hispanic (*n* = 24). Principals and special education teachers had equal education attainment levels spanning across college- and graduate/professional-level degrees.

**Table 1:** Principal and special education teacher characteristics.

	Principals (*n* = 26)		Teachers (*n* = 26)	
	n/*M*	%/*SD*	n/*M*	%/*SD*
Age	46.3	7.4	35.8	9.9
Gender				
Female	20	77%	24	92%
Male	6	23%	2	8%
Race/Ethnicity				
Asian	1	4%	0	–
Black	12	46%	2	8%
White Non-Hispanic	10	38%	24	92%
Latinx/Hispanic	4	15%	0	–
Education				
Bachelor’s degree	2	8%	2	8%
Graduate/Professional	23	88%	23	88%
Other	1	4%	1	4%
Years of experience	8.3	6.0	6.8	4.5

Race/Ethnicity reporting is non-exclusive, meaning summation of percentages is greater than 100%.

Twenty-six schools were represented in the sample. One school had one principal participate, but the teacher declined the interview due to lack of interest. Twenty-three schools had one teacher, and one school had two teachers in the sample. One principal and one teacher were excluded from this analysis, as they did not mention *autism-specific culture* in their interviews.

### Procedure

The senior author JL conducted individual, semi-structured interviews lasting 45–60 min with participating principals and special education teachers. Interviews were audiotaped and conducted at schools at a convenient time for participants. No field notes were made during interviews. Two parallel interview guides were developed using the Domitrovich et al. (23) multi-level framework. Questions were designed to elicit participants’ experiences with the EBP implementation process in their school, perceptions of the school environment, and behaviors and practices from other school staff (e.g., leadership, general education teachers, support staff) that had facilitated or hindered EBP implementation (see Supplementary Files S2, S3 for the principal and teacher interview guides, respectively). Example items from the principal interview guide include: “Tell me how you *facilitate* or *support* your special education teachers’ and classroom staff’s use of these practices.”; “Think about the autism support team at your school. Tell me about their relationships with the general education teachers and staff.” Example items from the special education teacher interview guide include: “What has it been like for you to implement EBPs in your classroom?”; “Tell me what makes it difficult to use these practices in your classroom.”; and “Tell me how the practices you use in your classroom fit within the school’s main goals and purpose.” Participants provided informed consent and were paid $50 for their time. The University of Pennsylvania IRB provided ethics approval for the study.

### Research team and reflexivity

The senior author JL is female, and at the time of the interviews, she was an assistant professor and had no previous relationship with the participants. Participants knew that the interviewer was a Ph.D.-level researcher with expertise in the clinical care of autistic children. As a licensed psychologist and implementation researcher, JL values the use of EBPs for autistic youth and supporting the successful implementation and sustainment of EBPs for autistic youth in public school settings. The remaining co-authors were not involved in data collection or initial qualitative data analysis. However, all authors are researchers committed to increasing access to best practices for autistic youth. Two authors (KA, AH) contributed to the thematic analysis of the autism-specific culture data; both identify as female and are in clinical psychology. The remaining authors contributed to manuscript writing and represent the following disciplines: special education (MH), clinical psychology (DT), and public health (TH). One co-author identifies as autistic and contributed their lived experience with the special education system and inclusive practices (CE).

### Data analysis

Semi-structured interviews were transcribed and uploaded to NVivo QSR 10 for data management. The coding scheme for the overall study was based in content analysis and developed using a systematic, rigorous, transparent, and iterative approach (41) and involved two stages. For Stage 1, the research team independently coded four transcripts to identify recurring codes and developed a preliminary codebook for principal and special education teacher interviews. As some codes were developed during the interview guide development and others arose from reading the transcripts, both a deductive and inductive approach were used (42). Two female research study coordinators with BA or higher degrees coded all data, and 20% of transcripts were selected randomly to calculate inter-rater reliability (43). Transcripts were randomly selected using a random number generator. Coders met regularly to discuss, clarify, and compare emerging codes and disagreements were discussed with the entire research team to reach consensus. Percent agreement was calculated based on the number of words agreed upon for Stage 1 coding. The average agreement for principal interviews was 97.04%, and for teacher interviews was 94.18%.

Stage 2 of the thematic analysis involved an iterative, inductive approach in which the segments of text related to autism-specific culture from Stage 1 were analyzed and coded to identify categories. The two female research study coordinators independently reviewed the segments of text from the autism-specific culture code to identify recurring themes (42). They met with the principal investigator (PI) to (a) develop a preliminary codebook integrating the identified themes, (b) operationally define each subcode, and (c) come to consensus on which subcodes to include in the final codebook. Lastly, the research study coordinators then coded all data, meeting regularly with the PI to discuss, verify, and compare subcodes and resolve any disagreements to attain consensus. Stage 2 inductive coding resulted in eight codes shared across principals and special education teachers with an additional two unique principal codes and two unique teacher codes. Codes were further organized into two broad themes regarding autism-specific culture. Specifically, the first theme was school staff’s characterization of their schools’ approach to inclusion, describing *inclusion philosophy*, *advocacy* for autistic students and the inclusion of these students, and ways in which autistic students are included (i.e., *academic inclusion* and *social interaction*). The second theme captured factors affecting autism-specific culture within schools, including facilitators of and barriers to supporting and including autistic students. For instance, both principals and special education teachers described *awareness* of and *attitudes* towards autistic students. Principals discussed *teacher/staff professional relationships* and their *[principal] involvement with autistic students* and special education teachers highlighted *teacher and staff support for inclusion* and *principal support for inclusion* as unique factors affecting autism-specific culture. Both principals and teachers identified *influences on inclusion* and the *location of the autism self-contained classroom* as components of autism-specific culture. Lastly, we also identified a third theme specific to special education teachers, including their recommendations regarding supporting autistic students and the inclusion of autistic students.

## Results

Results are presented by theme and integrate principal and special education teacher perspectives. Table 2 presents the code definitions, and Table 3 includes example quotes that illustrate the *autism-specific culture and inclusion* subcodes.

**Table 2:** Code definitions.

Code/subcode	Definition
Autism-specific culture and inclusion	Attitudes, perspectives, and treatment of autistic students in self-contained classrooms and their special education teachers; Inclusion (or lack thereof) in classrooms and other school spaces (e.g., lunchroom, assemblies) with neurotypical peers (e.g., integration or segregation of autistic students; active and supportive involvement from non-special education staff, such as general education teachers, principals, other staff)
Inclusion philosophy	Approach, guiding principles, decision-making for inclusion and integration practices
Advocacy	Non-specific sponsorship, support, and advocacy for autistic students and inclusion practices; on the part of the participant or others
Academic inclusion	Inclusion practices specific to academic spaces and activities (e.g., general education classrooms; fieldtrips with neurotypical peers; elective classes)
Social interaction	Non-classroom social inclusion and engagement with neurotypical peers (e.g., at recess, during lunch, non-academic clubs)
Awareness	Knowledge, understanding, awareness of autism; autism self-contained classrooms; autistic students’ needs and IEP goals (across greater school community, including general education teachers, staff, caregivers, neurotypical students)
Attitudes	Mode of thinking or feeling reflected in behavior toward autistic students in self-contained classrooms, teachers, classrooms; and inclusion and integration practices (across greater school community, including principals, general education teachers and students, staff)
Teachers/staff professional relationship (principal only code)	Special education teachers and general education teachers’ relationship with one another; helping each other, collaborating (e.g., planning fieldtrip together, working together on inclusionary practices); negative, poor, underdeveloped relationships
Teacher/staff support for inclusion (teacher only code)	Non-special education staff support and participation (or lack thereof) in inclusion and integration practices; cooperation and collaboration across teacher roles (e.g., general education teachers planning with special education teachers); disregard or negative regard towards inclusion practices (e.g., general education teachers ambivalent towards having autistic students in their classrooms)
Principal involvement with autistic students (principal only code)	Principal-specific involvement, interaction, and engagement with autistic students in self-contained classrooms
Principal support for inclusion	Principal-specific sponsorship, advocacy, and participation (or lack thereof) in inclusion and integration practices
Influences on inclusion	Specific determinants (i.e., barriers of and facilitators to) of inclusion and integration practices not captured in other subcodes
Location of self-contained classroom	Physical placement of self-contained classroom(s) in the school building
Recommendations	Expression or call for change and suggestions to improve inclusion and school culture

IEP, Individualized Educational Plan; EBP, evidenced based practice.

**Table 3:** Example principal and special education teacher quotes by subcode.

Subcode	Principals	Special education teachers
Inclusion philosophy	“It started with that, with talking about what inclusion truly is, not just doing it for the sake of doing it, but with doing it purposeful and having – making sure there’s an impact and making sure you can measure the growth.” [P3700; Principal, Female, Professional Degree]	“I get to be in a bubble… but there’s just so much going on outside of this bubble that we sort of get lost and left alone until there’s a problem.” [T217; Teacher, Female, Professional Degree]
Advocacy	“They know to call me because I need to see what this kid’s skill level is, and I need to see from the door what do we need to do with you, the kid… he needs to be mainstreamed. Make sure that happens.” [P500; Principal, Female, Professional Degree]	“I have a kid who is being mainstreamed pretty much the whole day and we came back from Easter break and then he did not wanna go back into the classroom at all. So I had to take over some of the reins myself where I was taking him around on my prep time and I was getting him back into the fray. I started out on the peripheral but then trying to phase [out my] support and get the one-on-one in there more.” [T801; Teacher, Male, Professional Degree]
Academic inclusion	“In smaller settings we identify different children, and we slowly try to include them more and more into the Gen Ed schedule. So, when those children are integrated into the whole group setting in a General Ed setting, they are often responded to very favorably.” [P4200; Principal, Female, Professional Degree]	“I wish there were more inclusion, but again, because there’s so many kids here with high needs… inclusion is really tough to do.” [T217; Teacher, Female, Professional Degree]
Social interaction	“Even if they are not ready for inclusion, maybe with their academics yet, then they at least get that social inclusion with age-appropriate peers.” [P900; Principal, Female, Professional Degree]	“The kids know our kids. They come in and they help. They do reading with the kids. They come in and they work with the kids. They’ll play on the computer with them. They’ll invite us to come in and do things. So although it seems as though we are a self-contained quiet little room, we are really not. We go out to recess with the kids. We go out to prep with the kids. And like I said, I interact with the other kids in the other class and my kids interact with those kids too. That’s the whole point of it.” [T921; Teacher, Female, Other Degree]
Awareness	“When you walk in our school, one of the first things you see is our pillar that says Autism Awareness… And so we want parents and community people to know that we have students with autism in our building and that we are aware and we are trying our hardest to meet their needs and include them as much as possible.” [P3700; Principal, Female, Professional Degree]	“People in this school, they are – what they know about autism is not really accurate… they are not really informed about the disorder, and I wanna bring autism awareness to the teachers in this school. Because I do not think everyone’s as informed as they should be.” [T3653; Teacher, Male, Professional Degree]
Attitudes	“[The autism self-contained classroom] just reinvigorates… because you do not get this all the time because it’s a struggle. It’s a daily struggle and sometimes it’s two steps forward and then five steps backwards with some of the kids. So, it’s just we feel very, if I can use the word blessed, to have our autistic children because as much as we feel that we have taught them, they have taught us so much more in so many ways that it’s amazing. It’s very gratifying as well.” [P5700; Principal, Female, Professional Degree]	“[General education] teachers are really afraid of kids with autism. They hear autism, and they are like, ‘Oh I cannot do that.’ And they do not understand that at least five of my kids are better behaved than a challenging general education student.” [T217; Teacher, Female, Professional Degree]
Teacher/staff professional relationships	“[Special education teachers] are including some of their children that are ready into regular Ed, so there’s a lot of conversations that are occurring between our regular Ed teachers… how to support those kids, what do we need to make it work?” [P5200; Principal, Female, Bachelor’s Degree]	N/A
Teacher/staff support for inclusion	N/A	“So my team meeting is actually with the other autistic support teacher. It’s not with the kindergarten teacher. So I really do not have any planning – common planning time with them. They’re both very friendly and I’ve talked to them, but very brief because we all have classes to attend to.” [T538; Teacher, Female, Professional Degree]
Principal involvement with autistic students	“So now we have breakfast every morning together just so that he could have a good day… and he told me today, ‘I love you, you are the best principal,’ and he says, ‘because you have breakfast with me every day’… So you know, those things are rewarding to see the progress in the students and knowing where he’s coming from, and knowing that I can make a difference in their lives… I try to keep that at the forefront.” [P2900; Principal, Female, Professional Degree]	N/A
Principal support for inclusion	N/A	“My principals really encourage the learning support teacher to buddy with the [special education] teacher and to make inclusion work that way of kind of being that bridge in between.” [T4257; Teacher, Female, Professional Degree]
Location of self-contained classroom	“Plus, their classrooms are on their age-appropriate floors and in their age-appropriate wing so that they get that exposure [to age-appropriate peers].” [P900; Principal, Female, Professional Degree]	“We’re on the fourth floor. We’re the only classroom up there. Um, we do have an art room and there is a media room. I’m not really sure what they do in that room but is pretty much just us on the fourth floor. So I kind of feel like we are isolated.” [T1828; Teacher, Female, Professional Degree]
Influences on inclusion	“They are included to the degree that they can, although also being respectful of some of their limitations, if there’s certain things that are not appropriate for them…if that was my child would not want them to be forced to do something just under the sort of umbrella of being included. So we try to be sensitive to that as well.” [P5700; Principal, Female, Professional Degree]	“We do not get to go out to recess with the kids because it’s just too many kids out there. The recess is K-5, so it’s just way too many kids and there’s only two or three staff members that monitor it and it’s overwhelming. So I think that part makes me really upset because I would love to see their interaction. And when we have had the chance, they play so well that I think their growth would be so much stronger if we had that interaction.” [T538; Teacher, Female, Professional Degree]
Recommendations	N/A	“It is little things like that, that could be addressed. You know, even if you put us on the second or third floor, I do not care, but at least, you know, I can go knock on a door next door just to say, ‘Hey, neighbor’… But we are just up there alone.” [T1828; Teacher, Female, Professional Degree]

Participant demographic characteristics and identification numbers are included in brackets; P, Principal; T, Special education teacher.

### Theme: approach to inclusion

#### Inclusion philosophy

Most principals described inclusion as “part of [their] vision” for autistic students in self-contained classrooms, such that they “want [autistic] students included wherever possible.” In expanding on their philosophy towards inclusion, some principals highlighted that they supported inclusion as “appropriate for [students’] growth,” such that students first need to “show that they are developmentally ready” for general education settings. Several principals described a goal of equality and a mission to treat their autistic students in self-contained classrooms “just like everyone else.” For instance, one principal stated that they were “trying to make it not be the Gen Ed population and [special education or autistic] population. It’s supposed to be, this is our [School name] population.” Another principal added that treating students equitably means having similar opportunities:

When I became an administrator, I was determined that there will be no special ed, gen ed. It’s just a school. So my expectations don’t waiver… everyone weighs in, different ways, but everyone is a part of it. My children with special needs are involved in any activity that we have at the school.

In addition, several principals described a need to be “purposeful” about inclusion, so “it’s just a part of what [they] do.” One principal added that there may need to be intentional strategies that facilitate inclusion, such as providing a student with limited language skills a whiteboard, so they have a way to communicate during a classroom discussion.

A few principals extended their inclusion philosophy to special education teachers, with one stating, “I want everyone to have relationships with everyone in my building. Because reality is we do not live in bubbles.” Another principal acknowledged that special education teachers can feel “alienated” from the rest of the school and, therefore wants them to feel like “a part of the fabric of the school.”

While one teacher specifically reported feeling included as “a part of the school,” half of teachers who referenced *inclusion philosophy* identified feelings of isolation. Teachers described being “secluded,” “left out,” feeling like “a lone wolf,” “in a bubble,” or even forgotten. One teacher summarized, “We often refer to ourselves as Special Ed Island. We’re off [isolated] and everybody else is their own thing” Another teacher stated that they do not feel intentionally left out, but they still end up on their own. In addition, some teachers described a lack of attention or prioritization of their autistic students or classrooms. For instance, one teacher noted that the school mission was created for general education students and, therefore, they had to “tweak it a little bit to work for [autistic students].” A few teachers described that their classrooms could follow along with the programming or curriculum of the rest of the school, and one teacher highlighted that there had been a recent “learning process” at their school in which the school is “coming around” to inclusion, such that each year they are being included in more activities than the previous year.

#### Advocacy

About half of principals (56%) outlined ways in which they advocate for their autistic students and special education teachers. Several described “pushing for” inclusion and advocating for their students to have increased time in general education settings. Principals reported conducting classroom observations, setting individualized student goals, obtaining tools to support progress monitoring of included students, and working closely with special education teachers, who they describe as the strongest advocates for autistic students, to facilitate inclusion. Additional strategies that principals employed include purchasing materials to support the inclusion of autistic students in activities (e.g., noise canceling headphones, multisensory equipment) or advocating for additional staff at the district level.

Forty percent of teachers commented on ways in which they advocate for their autistic students or strategies that they feel worked well for autistic students (e.g., positive behavioral supports). Several teachers noted that they go out of their way to get to know general education teachers and have ongoing conversations about inclusion. One teacher described that they needed to be the driver of inclusion: “If I ask to be included, like I say… “Oh, we would really enjoy going on [field] trips with you.” “Of course,” they say, “absolutely.” And they have included us in those [field] trips.” Other teachers described their additional efforts to advocate for inclusion, such as taking their students to different general education art classes or encouraging students to sit with their autistic students at lunch.

#### Academic inclusion

Principals and special education teachers described what academic inclusion looks like for their autistic students in self-contained classrooms. Most principals (92%) detailed the academic inclusion of autistic students. Overwhelmingly, principals said that autistic students in self-contained classrooms have “as much inclusion time as possible,” though principals typically qualified this statement to indicate that the level of inclusion “depends on the child.” One principal summarized, “Now not all kids can, but if they can, we mainstream them.” Principals reported a wide range of what inclusion looks like in their schools from autistic students participating in general education specials, such as art, to full inclusion. For instance, one principal described,

We look at every kid and make sure they’re getting the proper programming. We actually have [autistic] kids in our building who are not in [a self-contained] classroom at all and they never have been… And we say, all right, well, if we can do X, Y, and Z, we’ll keep it going.

Principals also described various factors that support the academic inclusion of autistic students in their schools. For instance, some principals highlighted the importance of communication amongst teachers, with one principal noting that they have developed “a really good system of students passing in and out of classrooms,” such that autistic students spend time in general education and general education students may receive support from the special education teacher. Relatedly, another principal gave an example of how teacher involvement supports academic inclusion:

At that IEP meeting, the regular teacher was there with the parent, she was able to clearly speak to the parent about the child, where the weaknesses were, where her strengths were. So that makes parents feel good when a regular ed teacher comes in and treats your child like they’re a member of their class. They don’t see them as the girl in the [self-contained] class.

In addition, other principals emphasized the importance of a systematic approach to inclusion, such as starting gradually (e.g., one academic class) or using progress monitoring to monitor autistic students’ IEP goals when in inclusive classrooms.

Of the special education teachers who mentioned academic inclusion (64%), a substantial minority (5 of 16) stated that they would like to see more inclusion of their students. One teacher described, “I feel like the inclusion process is not necessarily inclusive just because my kids are in the same space during [specials]… when they go to gym class, they do not do the stuff that the other kids are doing.” Another teacher added, “At the beginning of the year I was told that my students would go on field trips with the first-grade classrooms. That has never happened.” In contrast, some teachers noted examples of their autistic students being integrated into general education classrooms. One teacher described their school as “like Grand Central Station,” such that students are frequently being “pushed in” to classrooms and coming and going from different classrooms. Another teacher highlighted that many of their students are academically ready for inclusion but other factors (e.g., behavior) interfere.

#### Social interaction

Principals and special education teachers identified social (peer) interaction as another aspect of inclusion for their autistic students in self-contained classrooms. Principals discussed ways in which they facilitated peer interaction for autistic students, noting that if autistic students were not ready for academic inclusion “then they at least get that social inclusion with age-appropriate peers.” For example, several principals identified lunch and recess as important opportunities for autistic students in self-contained classrooms to spend time with their general education peers, with one principal changing the schedule to facilitate autistic students joining recess with the general education kindergarten class. Another principal described “reverse inclusion”:

In the lunchroom, the children from the typical classrooms are sitting with the children—autistic students—at their lunch tables, and they’re just interacting and socializing. And the adults are helping to facilitate that when it doesn’t come naturally. But for some, it’s coming naturally. So that’s been helping with the social skills.

Several principals emphasized that peer support is valuable for both general education students and autistic students. For instance, one principal described middle schoolers who were on the same floor as the self-contained classroom building “positive relationships” with the autistic students and volunteering in the classroom. Other principals noted partnerships between the autistic self-contained classroom and other grade level classrooms, including pairing students for specials or for joint field trips. One principal added that they give “friendship awards” for students “who have volunteered their lunch time to play and socialize with our [autistic] students.” This principal described an increase in the collaborative spirit in the school, such that, “It’s really nice to see the kids who have rallied around and taken that child under their wing to help them, to make sure that their transitions are smooth and that they do not get upset.”

Thirty-two percent of teachers referenced social interaction with mixed descriptions in how often autistic students in self-contained classrooms were socially included with their general education peers. However, overall, teachers agreed regarding the importance of social opportunities for their autistic students, with one teacher stating, “The academic part is important, but also there needs to be a balance of what can they learn socially, and how can they learn with speaking to one another.” Another special education teacher who lamented the lack of inclusion for their students said, “I think their growth would be so much stronger if we had that interaction,” referencing joint recess. Other teachers reported positive social interactions between students, highlighting shared recess, lunch, and joint classroom time as opportunities for interaction. Teachers also described buddy systems in which general education students would come into self-contained classrooms to work with the autistic students or autistic students would join their grade level peers’ classrooms at the end of the day for “[free] choice time.”

### Theme: factors affecting the inclusion of autistic students in self-contained classrooms

#### Awareness

While fewer than half of principals (44%) referenced awareness of autism and the autism self-contained classrooms in their schools, awareness was the most referenced topic (88%) by teachers. Of the principals who mentioned awareness, a few highlighted ways in which autism awareness is a value of their schools. For instance, one principal described specific autism awareness activities (e.g., selling t-shirts) at their school. Another noted that “almost all” of the school was aware of the autism self-contained classrooms, from parents to students to custodial staff. In addition, one principal stated that certain school practices, specifically creating grade level (e.g., Kindergarten to second grade) “communities,” facilitated awareness of their autistic students and classrooms as “everybody [within the community] knows how everybody else operates.” In contrast, some principals indicated that autism awareness is an area for growth in their schools, as only certain school staff (e.g., special education team, speech-language or occupational therapists) are aware of the autism self-contained classrooms.

Similarly, special education teachers identified specific school staff who may be aware of the autism self-contained classrooms in the school; however, the majority of teachers who mentioned awareness (81%) cited at least one misconception from colleagues regarding autism, the abilities of their autistic students, or the strategies used in their classrooms. For instance, one teacher described:

I don’t think that the rest of the staff necessarily understands what autism is or what it means for my kids…I think people are kind of just not holding them to the same kind of expectations in their [inclusion classes]… because people don’t understand what autism is and that it’s not necessarily an intellectual disability… I just wish that there was a little more awareness or training for our school staff.

Another teacher expressed upset at the implications of school staff’s misconceptions and stigma of autism or students’ potential:

These are people that are in a teaching environment… And they can't understand it. So how is someone in the public supposed to understand what's going on? And I think it is harder with autistic children because they don't have [the] physical features that other children with special needs have… And it's hard to differentiate that when you're out in the public or in the hallway. They don't realize.

Relatedly, several teachers noted that general education teachers express surprise at the skill level or work accomplished by autistic students in self-contained classrooms, potentially because it did not match their preconceived stereotypes of autism. One special education teacher added that while their colleagues may notice and commend them on their students’ behavioral gains, they do not notice autistic students’ academic achievements:

People will stop me in the hallway and be like, “Oh, your kids line up now, and they never used to do that before. That’s great.” … It’s really only what they see, where I don’t think that their academic progress is on anyone’s radar besides mine.

When asked directly about general education teachers’ awareness of the autism self-contained classrooms or strategies used, a common response from special education teachers was, “They do not know what I do.” Moreover, several teachers described their colleagues’ misperceptions of their jobs as “play[ing] all day long” or being “easy.” Further, special education teachers indicated that it is challenging when their colleagues do not understand the strategies or purpose of the strategies employed in the autism self-contained classroom. For instance, one teacher described, “They’ll [colleagues] say, ‘Why is your room so dark? It needs to be brighter.’ I’m like ‘Well, it really causes sensory overload for a lot of the students to keep it so bright.’”

#### Attitudes

Nearly half of both principals (48%) and special education teachers (40%) referenced attitudes towards autistic students in self-contained classrooms or inclusion of these students. Several principals referenced that they “embrace” the inclusion model and are supportive of the autistic students in their school. One principal described that it is important to be aware and accept the differences of autistic students compared to non-autistic students, stating:

Understanding that it may look very different than a typical first grade when you go in because the kids… they may be louder. They may need more transitions. The teacher may do things that may look a little different. But I think understanding that they’re trying to meet the needs of their kids… I think it’s just accepting that, as well.

A few principals acknowledged that this attitude is not universal, as school staff might become frustrated by specific students’ behavioral challenges. However, many principals referenced general education students in their building being friendly, tolerant, and motivated to “help one another and be supportive.”

Special education teachers echoed that students in general education settings largely had positive attitudes toward autistic students. However, teachers reported that while some general education teachers were respectful of their work, many had negative attitudes towards inclusion and practices used in autism self-contained classrooms. In a few instances, special education teachers discussed hearing exclusionary comments from colleagues such as, “They’re your problem,” and, “Your students cannot go into my room.” In addition, some teachers were described as “afraid” of autistic students, sometimes due to interfering behaviors (e.g., biting, hitting), though several special education teachers attributed others’ fears to lack of knowledge or understanding. For instance, one participant noted a shift in attitude resulting from increased exposure to working with autistic students:

I think they [general education teachers] were scared at first. And then once they got to know the kids, they loved them. I mean, they’re easy to love. So, I just – I feel like they were – they didn’t know what to do at first. And then once they got to know them, everything started changing.

General education teachers also reportedly expressed resistance to strategies used by special education teachers, most prominently positive behavior reinforcement models, or the supports in place for the autism self-contained classrooms. One teacher stated that other teachers see their role as a “babysitter” and do not understand why they have additional staffing in their classroom.

#### Teacher/staff professional relationships (principal code) and teacher/staff support for inclusion (teacher code)

Most principals (76%) described ways in which teacher/staff relationships affect the autism-specific culture in their schools. Principals consistently highlighted communication, including formal and informal opportunities, as being helpful for supporting and including autistic students “so everybody’s on the same page.” Communication occurs across educator roles, though principals predominantly discussed the importance of communication between special education and regular education teachers. For instance, one principal described:

[Special education teacher] works with [general education teacher] to provide the appropriate levels of support and understanding what they’re doing so he can support [autistic students] when they come back in the [autism self-contained] classroom. And so really communicating around what the needs of kids are and how they can incorporate them into what they’re doing in the regular education classroom so that he can incorporate that.

Principals noted that professional learning communities, grade group meetings, and other meetings as opportunities during which teachers and the principal can discuss student progress. One principal summarized:

I think there’s constant conversation and discussion about those students. It’s not just, “Okay, your side’s here, have a good year.” There’s constant community and discussion about those students, how well they’re doing, what their needs are, what they’re doing well, what they need support with.

Some principals observed poor relationships across roles, sometimes stemming from navigating interfering student behavior. Others noted limited formal time communication when structures are not in place for regular meetings; for example, there is not a regular meeting set up for special education teachers to communicate with other teachers.

Teacher/staff support for inclusion was one of the most frequently referenced (84%) domains by special education teachers. Teachers’ perspectives were mixed in terms of their experiences of support in their schools. Several special education teachers indicated that supportiveness depended on the “comfort level” of the individual, and experiences ranged from positive to negative to ambivalent. While some teachers referenced being welcomed into general education teachers’ classrooms, others described that they are just “on an Island by myself,” with one special education teacher sharing that when they provided coverage for a classroom, they were mistaken as a substitute. Another teacher specifically summarized their experience of using positive behavioral supports in their autism self-contained classroom:

I get a lot of flak from my coworkers and even at times from – not our principal, but other administrators because I don’t use that negative punishment model. I use a positive behavioral support model… I get a lot of eye-rolling, I get a lot of you’re too soft from people.

In addition, participants described valuing opportunities to have shared meetings with their same grade colleagues. However, scheduling was a tremendous barrier to support from other teachers. Though some special education teachers had brief meeting times, most said they were not given shared meeting times with their grade groups, felt left out during planning, or that shared meetings covered topics not applicable to autism self-contained classrooms.

#### Principal involvement with autistic students (principal code) and principal support for inclusion (teacher code)

Twenty percent of principals discussed ways in which they are involved with their autistic students or autism self-contained classrooms. Principals highlighted how they develop relationships and rapport with their students, including having breakfast with an autistic student and visiting and observing the autism self-contained classrooms. Some principals specifically described ways in which interacting with autistic students facilitates inclusion. For instance, one principal shared that they talk to all newly enrolled special education students, stating staff “know to call me because I need to see what this kid’s skill level is, and I need to see from the door what do we need to do… [if he/she/they] needs to be mainstreamed.” Another described their observations as key to identifying students for inclusion.

Of the special education teachers who referenced principal support for inclusion (32%), participants described both supportive and unsupportive administrations. Support included cultivating an inclusive environment through including autistic students in schoolwide events (e.g., assemblies), observing in the autism self-contained classroom, and encouraging teacher communication to be a “bridge in between” classrooms. Teachers who described unsupportive environments mostly highlighted being left alone. For instance, one teacher stated, “I do not get chastised by administration or asked to change my methods, I just do not get a lot of support.” Other participants indicated that they would like the principal to be more participatory in their classrooms, including more frequent observations and getting to know the autistic students, and to facilitate teacher communication.

#### Location of autism self-contained classroom

Some of the principals (24%) and teachers (20%) highlighted the location of the self-contained classroom as either a facilitator of or barrier to the inclusion of autistic students. For instance, a few principals highlighted being intentional with classroom location, such as having autistic students in the “same hallway” as their general education peers. One principal described:

Before I got here, it was like the special ed wing, like all special ed [classrooms] are in one area. And I said that was the first thing I needed to change in terms of putting all the different classes within the flow of the school and not separating them into one part of the building and acting like they’re not there…That it’s not like they’re going through the back door. They’re going through the same doors everyone else can.

Another principal referenced space issues as contributing to the autistic students being separated from their grade level peers. Notably, all the teachers who mentioned their classroom location indicated that they were “secluded,” with one teacher stating, “We’re just stuck over here in nowhere land by ourselves.” Other teachers described that their self-contained classrooms were on a separate floor (e.g., basement, fourth floor) where there were no other classrooms.

#### Influences on inclusion

About half of principals (44%) and special education teachers (48%) noted factors that influence inclusion that were not captured elsewhere. Principals predominantly discussed facilitators of and barriers to inclusion specific to student characteristics and teacher/staff factors. Specifically, some principals noted that students’ skills, including their communication, academic skills, and interfering behavior, affect inclusion. For instance, one principal stated that autistic students who are behind their peers in certain subjects “are not able to fit into the [academic] group[s] that the teachers have already established.” Another principal noted that an autistic student’s behavior had “become a danger to himself and others” and, therefore, they included him in classes in which they were less likely to see interfering behaviors. In addition, principals described ways in which teacher/staff factors affected inclusion. Principals noted that the personalities (e.g., kindness, calm) and techniques of teachers facilitated the inclusion of autistic students. For instance, one principal stated, “I have seen some positive responses from teachers and implementation of specific techniques and that has afforded us opportunities in some instances to include students.” Several principals highlighted the value of having additional staff, such as a one-to-one aide or classroom assistant, to accompany autistic students between classrooms (i.e., general education and autism self-contained classrooms). One principal specifically referenced a district-level factor (i.e., lack of hiring) as a barrier to supporting the inclusion of autistic students.

Similar to principals, special education teachers also cited student and teacher/staff factors as influences on inclusion. Some teachers noted that some of “their autistic students are “academically on par and ready to be there [included in general education],” but their “behaviors” (e.g., tantrums) interfere. One teacher’s perspective was that “functionally [their] students cannot do it [inclusion]” or referenced the “high needs” of the students as a barrier to inclusion. Other teachers emphasized that a facilitator of inclusion was having a classroom assistant or aide to support autistic students in settings in which they are included, though several teachers noted that their schools simply did not have the staffing for this. One teacher described that lack of staff support meant that it would be a tradeoff, such that if the classroom assistant accompanied the autistic students into general education classrooms, the special education teacher would then be the only adult in the autism self-contained classroom. Beyond staffing, participants also described lack of funding as a barrier to inclusion, such as not being granted funds for a bus for autistic students to join field trips. Another teacher summarized, “Trying to do a co-teaching model of inclusion is a big jump. It’s huge. And I do not think anybody has the time, energy, or resources to head it up.”

### Theme: recommendations

A subset of special education teachers (36%) provided recommendations, which fell into three categories: specific recommendations for inclusion, recommendations for teachers, and recommendations for administration. In terms of inclusion, teachers primarily wanted their autistic students to receive more time in inclusive settings in general, whether this was an integrated classroom, shared recess time, or more integration during lunch time. One teacher noted that having more inclusion time also would facilitate collaboration between teachers. For instance, a participant stated:

If I was included in grade group or – I think that would be very helpful. Then they would know what I was doing, and they would know how our schedule works, and they would understand it better. But we don’t get the time at all to collaborate with the teachers.”

For general education teachers, special education teachers recommended them spending more time getting to know the autistic students and treating them equitably. Behavior management training also was recommended, particularly for behaviors that interfere with inclusion. Administrative recommendations included moving the special education classroom, so it is not physically isolated, keeping class sizes small, supporting autism-specific staff development to reduce stigma and build skills specific to service autistic students, funding more classroom staff, and giving an equal amount of support to specialized classrooms as general education classrooms.

## Discussion

The findings of this qualitative study shed light on the culture of inclusion of autistic children in public elementary schools in the United States from the perspectives of principals and special education teachers. In line with previous research [e.g., (9, 11, 12, 44, 45)], this study emphasized that inclusion goes beyond placement and academic integration alone, and revealed possible attitudes and stigma toward autism that may affect inclusion. Participants emphasized the need for proactive measures to facilitate the genuine and equitable social participation of autistic students in a manner that fosters positive experiences for them, with a specific focus on improving knowledge and attitudes toward autistic students. It is important to note that educators did not endorse one universal picture of autism in schools, nor did they propose one specific strategy to promote inclusion. Instead, our findings highlight that there are several different ways to be inclusive and that schools must take steps to promote inclusion in a way that is personalized for the unique needs of their setting, staff, and autistic students. Implications for how to support the inclusion of autistic students with a diverse range of strengths and support needs are described below.

A consistent theme that emerged from both principals and special education teachers was a strong desire for equality for autistic students in terms of similar physical placements and social and academic opportunities. Notably, a small group of principals also emphasized the importance of *equity* (i.e., each person has different circumstances and requires a different set of resources and opportunities to reach an equal outcome) as opposed to equality [i.e., each individual or group of people is given the same resources or opportunities (46)] in promoting inclusion, providing insight on an “ideal” when it comes to the culture of supporting autistic students in school. These participants emphasized that true inclusion requires a nuanced understanding of individual student needs and the provision of appropriate and individualized support. Going forward, inclusion efforts should not solely aim to treat all students equally, but rather create inclusive environments that equitably meet all students’ needs and ensure autistic students’ meaningful participation in the educational setting.

This study also confirmed the stigmatization experienced by autistic students in schools (10, 47–49). Participants cited autistic students’ behaviors as one factor that interfered with inclusion. In the school environment, autistic children display behaviors, such as tantrums, aggression, and not following directions, at a higher rate than their neurotypical peers (50–52), which can be a driver of stigmatizing views. This stigmatization can contribute to the misperception that inclusion is only for “some” student and not “all,” which further impedes achieving true inclusion. Our findings also revealed an additional layer of stigmatization experienced by special education teachers themselves, similar to “affiliate stigma” of parents of autistic children (17), highlighting the challenges they face in promoting inclusive practices. This mutual experience of stigmatization underscores the complexity of creating an inclusive environment and emphasizes the need for a comprehensive approach to promoting a culture of inclusion that addresses the systemic barriers faced by both autistic students and teachers. Participants in the study recommended additional training on disabilities and special education, as well as steps to promote awareness of disabilities and special education in schools to help reduce stigma, as school staff’s attitudes have been shown to be barriers to inclusion (26). Additionally, research has found that interventions including psychoeducation, case vignettes, contact-based interventions, and bespoke stigma reduction interventions have helped to reduce stigma surrounding both autism and other developmental and mental health concerns in educational settings (53).

These themes support the overarching philosophy and goals of the IDEA and, more specifically, the expanded definition of the “least restrictive environment” for autistic students in educational settings in the last several decades, now being viewed as more of a “context” than a “place” (54, 55). Additionally, these views of inclusion parallel the perspectives of many autistic self-advocates themselves (see the aforementioned definition of inclusion by our co-author, CE). However, these models and ideals are still not widely applied throughout the public education system in the United States.

### Practical recommendations

A key suggestion to promote inclusion put forward by participants is the implementation of a co-teaching model involving both special education teachers and general education teachers in the same classroom. Previous research has highlighted that among general education teachers, those who possess limited knowledge or training in special education tended to engage in inclusive practices less frequently (56). A more collaborative teaching approach may allow for the sharing of expertise, resources, and responsibilities, fostering a more inclusive learning environment for all students. However, it should be noted that although participants in the study stated that co-teaching would be a practical step to promote true inclusion, they also reported that actually initializing this model would be a “big jump.” Previous research has highlighted similar educator attitudes in regard to initiating a co-teaching model; however, several studies have found positive administration support and specified professional training as helpful strategies to promote successful implementation (57–59).

In previous work in this area, teachers have frequently highlighted the absence of adequate training and resources as a major obstacle to establishing an inclusive classroom atmosphere and effectively addressing behaviors of autistic students that may interfere with classroom instruction (26, 60–62). The need for additional training in autism, inclusive practices, and inclusive teaching methods also was identified in the current study as a valuable strategy to promote inclusion. By enhancing educators’ knowledge and skills in these areas, attitudes towards inclusion can be positively influenced, contributing to a more inclusive school culture overall (26, 63). An essential next step to promote inclusion is investing in professional development opportunities that address the specific needs of educators working with autistic students.

Educators in this study also highlighted the positive attitudes of neurotypical students at their school towards interacting with and supporting autistic students. School staff could capitalize on this openness by educating neurotypical students about neurodiversity and promoting positive and collaborative interactions with their autistic peers. Examples of this may include partner or group academic activities or setting up relationship building activities either during unstructured school time or after school extracurriculars (64). These approaches have the potential to create an inclusive school culture that values and celebrates differences.

Moreover, educators shared ways in which they consider individualizing educational support for autistic students’ strengths and challenges in various academic, social–emotional, and behavioral realms in relation to “readiness” for inclusion. However, this notion somewhat contradicts the essence of true inclusion, which emphasizes providing the necessary support and accommodations to enable autistic individuals with a diverse range of strengths and support needs to succeed in the general education setting. The findings highlight the need to challenge and shift this narrative within schools, encouraging a paradigm that values neurodiversity and focuses on providing the appropriate support and models for autistic individuals to succeed in inclusive settings, rather than imposing readiness criteria that may hinder their inclusion (65). Approaches such as Universal Design for Learning (66, 67) and collaborative teaming (68, 69) have been used to create inclusive settings that can accommodate and serve all children. Additionally, behavioral interventions [e.g., RUBIES (70)] can help educators manage behaviors that they report prevent some autistic students from fully engaging in an inclusive classroom.

### Limitations and future directions

It is important to note that this study focused specifically on the perspectives of elementary school principals and special education teachers in one geographic area in the United States. Future research should consider incorporating the viewpoints of other stakeholders, most importantly autistic students themselves, from more geographically diverse areas of the country. It also will be critical to survey stakeholders involved in middle and high school education for autistic students given the changes and challenges that occur in adolescence. Exploring these diverse perspectives will contribute to a more comprehensive and generalizable understanding of the culture of autism in schools and help inform the development of truly inclusive practices that consider the needs of a broader group of autistic individuals and their educators. In addition, while consistent with observed trends in public schools across the United States, the majority of principals and special education teachers in this study were female.

## Conclusion

This study offers a window into educators’ perspectives on and recommendations for improving inclusion in schools with self-contained settings and provides valuable insights for policymakers, school administrators, educators, and other professionals involved in the education of autistic students. To promote true inclusion, it is crucial to prioritize equity over equality, recognize and address social as well as academic inclusion, combat stigmatization of both autistic students and special education teachers, challenge readiness-based narratives, and embrace individualized approaches to support diverse learners. By doing so, schools can foster inclusive environments that celebrate neurodiversity and create opportunities for the academic, social, and emotional growth of all students.

## Data availability statement

The raw data supporting the conclusions of this article will be made available by the authors, without undue reservation.

## Ethics statement

The studies involving humans were approved by University of Pennsylvania IRB. The studies were conducted in accordance with the local legislation and institutional requirements. The participants provided their written informed consent to participate in this study.

## Author contributions

JL is the principal investigator of the study, generated the idea and designed the study, and supervised the qualitative coding and data analysis. KA is the primary writer of the manuscript and summarized the qualitative data. MH, DT, CE, AH, and TH supported the writing of the manuscript. All authors contributed to the article and approved the submitted version.

## Funding

This manuscript was supported by the following grants from the US National Institute of Mental Health: K01 MH100199 (Locke) and the Health Resources Services Administration (HRSA) of the U.S. Department of Health and Human Services (HHS) Autism Intervention Network on Behavioral Health (AIR-B; UT3MC39436; Locke, Ahlers). The information, content and/or conclusions are those of the authors and should not be construed as the official position or policy of, nor should any endorsements be inferred by HRSA, HHS or the U.S. Government. AIR-B is funded through a cooperative agreement with HRSA/MCHB. The funders had no role in the design of this project, in the writing of the manuscript, and in the decision to submit this manuscript for publication.

## Conflict of interest

The authors declare that the research was conducted in the absence of any commercial or financial relationships that could be construed as a potential conflict of interest.

## Publisher’s note

All claims expressed in this article are solely those of the authors and do not necessarily represent those of their affiliated organizations, or those of the publisher, the editors and the reviewers. Any product that may be evaluated in this article, or claim that may be made by its manufacturer, is not guaranteed or endorsed by the publisher.
