# Supplementary material for: “On an island by myself”: implications for the inclusion of autistic students in self-contained classrooms in public elementary schools
Source: Front Psychiatry. 2023 Sep 26;14:1241892. doi: 10.3389/fpsyt.2023.1241892 (PMC10566509; doi:10.3389/fpsyt.2023.1241892)
Supplement: Supplementary file 1 [file Data_Sheet_1.docx]

Supplementary Material

“On an Island by Myself”: Implications for the Inclusion of Autistic Students in Self-Contained Classrooms in Public Elementary Schools

Kaitlyn Ahlers,^1,2^ Maria L. Hugh,^3^ Daina Tagavi,^4^ Curtis Eayrs,^5^ Alyssa M. Hernandez,^6^ Theodore Ho,^7^ and Jill Locke^5*^

*** Correspondence:** Corresponding Author: jjlocke@uw.edu

# COREQ

**COREQ (COnsolidated criteria for REporting Qualitative research) Checklist**

A checklist of items that should be included in reports of qualitative research. You must report the page number in your manuscript where you consider each of the items listed in this checklist. If you have not included this information, either revise your manuscript accordingly before submitting or note N/A.

| Domain 1: Research Team and Reflexivity | Guide questions/description | Present |
| --- | --- | --- |
| Personal Characteristics |  |  |
| 1. Interviewer/Facilitator | Which author/s conducted the interview or focus group? | Y |
| 1. Credentials | What were the researchers’ credentials? E.g., PhD, MD | Y |
| 1. Occupation | What was their occupation at the time of the study? | Y |
| 1. Gender | Was the researcher male or female? | Y |
| 1. Experience and training | What experience or training did the researcher have? | Y |
| Relationship with Participants |  |  |
| 1. Relationship established | Was a relationship established prior to study commencement? | Y |
| 1. Participant knowledge of the interviewer | What did the participants know about the researcher? E.g., personal goals, reasons for doing the research | Y |
| 1. Interviewer characteristics | What characteristics were reported about the interviewer/facilitator? E.g., Bias, assumptions, reasons and interests in the research topic | Y |
| Domain 2: Study Design |  |  |
| 1. Methodological orientation and Theory | What methodological orientation was stated to underpin the study? E.g., grounded theory, discourse analysis, ethnography, phenomenology, content analysis | Y |
| 1. Sampling | How were participants selected? E.g., purposive, convenience, consecutive, snowball | Y |
| 1. Method of approach | How were participants approached? E.g., face-to-face, telephone, mail, email | Y |
| 1. Sample size | How many participants were in the study? | Y |
| 1. Non-participation setting | How many people refused to participate or dropped out? Reasons? | Y |
| 1. Setting of data collection | Where was the data collected? E.g., home, clinic, workplace | Y |
| 1. Presence of non-participants | Was anyone else present besides the participants and researchers? | Y |
| 1. Description of sample | What are the important characteristics of the sample? E.g., demographic data, date | Y |
| Data collection |  |  |
| 1. Interview guide | Were questions, prompts, guides provided by the authors? Was it pilot tested? | Y; No pilot testing |
| 1. Repeat interviews | Were repeat interviews carried out? If yes, how many? | N; No repeat interviews |
| 1. Audio/visual recording | Did the research use audio or visual recording to collect the data? | Y |
| 1. Field notes | Were field notes made during and/or after the interview or focus group? | Y |
| 1. Duration | What was the duration of the interviews or focus groups? | Y |
| 1. Data saturation | Was data saturation discussed? | Y |
| 1. Transcripts returned | Were transcripts returned to participants for comment and/or correction? | N; No return of transcripts |
| Domain 3: Analysis sand findings |  |  |
| 1. Number of coders | How many data coders coded the data? | Y |
| 1. Description of the coding tree | Did authors provide a description of the coding tree? | Y |
| 1. Derivation of themes | Were themes identified in advance or derived from the data? | Y |
| 1. Software | What software, if applicable, was used to manage the data? | Y |
| 1. Participant checking | Did participants provide feedback on the findings? | N; No participant checking |
| Reporting |  |  |
| 1. Quotations presented | Were participant quotations presented to illustrate the themes/findings? Was each quotation identified? E.g., participant number | Y |
| 1. Data and findings consistent | Was there consistency between the data presented and the findings? | Y |
| 1. Clarity of major themes | Were major themes clearly presented in the findings? | Y |
| 1. Clarity of minor themes | Is there a description of diverse cases of discussion of minor themes? | Y |

Developed from: Tong A, Sainsbury P, Craig J. Consolidated criteria for reporting qualitative research (COREQ): a 32-item checklist for interviews and focus groups. *International Journal for Quality in Health Care*. 2007. Volume 19, Number 6: pp. 349 – 357

**Once you have completed this checklist, please save a copy and upload it as part of your submission. DO NOT include this checklist as part of the main manuscript document. It must be uploaded as a separate file.**

# Principal Interview Guide

**School District of {City} Principal Interview**

**Reminder of consenting to recording**: We just want to reconfirm that we have your permission to audio record this interview. All information will be completely confidential and the recordings will be deleted after we have transcribed the interview.

***If phone interview***: For the purposes of the recording, we ask that you not put us on speaker phone as it will degrade the quality of the audio. We also ask that you close the door to ensure your privacy and confidentiality.

[Turn on Recorder]

**Instructions:** The School District of {City} has arranged for autism [self-contained classroom] staff to get training in several evidence-based practices including discrete trial training, pivotal response training, functional routines, positive reinforcement, and visual schedules. We would like to learn from you about your experiences using these practices.

Do you have any questions before we get started?

1. Tell me what it is like for you to work in the School District of {City} now.
2. Tell me about your school environment. **[Clarify: what it is like as a place to work].**
   1. What is a typical day like for you?
   2. How do the practices intended for your autism [self-contained] classrooms fit within the school’s main goals and purpose?
      1. **[If they do not know about the practices]** In what ways could you be more involved in the process?
   3. What would help you be more involved in the process [in the future]
   4. How would you describe your school’s relationship with the surrounding community?
3. Think about the autism [self-contained classroom] team at your school. Tell me about their relationships with the general education teachers and staff.
   1. **[If they are involved]** Tell me how the general education teachers and staff are involved.
   2. **[If they are not involved]** How would you involve general education teachers and staff with your autism [self-contained] classrooms?
      1. What would this look like?
4. Who else in your school knows about what your autism [special education] teacher and classroom staff are using in their classroom?

***[(POTENTIAL PROBES)]***

- 1. Tell me how other teachers in your school support the use of these practices in the autism [self-contained] classrooms.
  2. Tell me how your Special Education Liaison (SEL) supports the use of these practices in the autism [self-contained] classrooms.

1. Tell me how you *facilitate* or *support* your autism [special education] teachers’ and classroom staff’s use of these practices.
2. How do you support your autism [special education] teachers and classroom staff in learning more about these practices?
3. In what ways do you recognize, appreciate, or reward your teachers’ and classroom staff’s use of these practices with their students with autism?
4. What would help you support your teachers and staff use these practices more effectively?
5. Tell me about the most recent time you visited your K-2 autism [self-contained] classroom.
6. How do the autism [special education] teacher and the support staff interact with one another?
7. What happened the last time you visited that classroom?
8. In what ways were you knowledgeable about the practices that were being used in that classroom?
9. What support could you use in the future to help the teachers with their use of these practices?

***[(POTENTIAL PROBES)]***

1. Can you talk more about that?
2. What feedback did you provide to the teacher and classroom staff on their use of these practices during your visit?

***[(POTENTIAL PROBES)]***

1. How was feedback delivered to the classroom team?
2. How did the classroom team respond to that feedback?
3. **[If no feedback was provided]** Tell me more about that decision.
   1. What support or training do you need to give constructive feedback in the future?
4. Think of the last time that you had a meeting with your autism [special education] teacher and/or classroom staff? Tell me what happened.
5. How often do you meet with them?
6. What do you hope to accomplish with these meetings in the future?
7. Think of a recent challenge or barrier that your autism [special education] teacher and/or classroom staff had when using one of these practices in their classroom.
8. What was that challenge or barrier? **[PROBE: Can you tell me more?]**

- **[If cannot recall any challenges]** Tell me what the autism [special education] teacher and/or classroom team does to avoid challenges to implementation of these practices.
  - 1. Tell me what you do to prevent implementation challenges from occurring.

1. Tell me the steps that you took to help remove or address that barrier.
2. What strategies could be used to address barriers in the future?
3. **[If cannot name any strategies]** What might help you better understand these challenges that your teachers and classroom staff face and/or help them with these challenges?
4. What else would you like to add that I have not asked you?

**Thank you so much for your time!**

# Special Education Teacher Interview Guide

**School District of {City} Teacher Interview**

**Reminder of consenting to recording**: We just want to reconfirm that we have your permission to audio record this interview. All information will be completely confidential and the recordings will be deleted after we have transcribed the interview.

***If phone interview***: For the purposes of the recording, we ask that you not put us on speaker phone as it will degrade the quality of the audio. We also ask that you close the door to ensure your privacy and confidentiality.

[Turn on Recorder]

**Instructions:** The School District of {City} has arranged for autism [self-contained classroom] staff to get training in several evidence-based practices including discrete trial training, pivotal response training, functional routines, positive reinforcement, and visual schedules. We would like to learn from you about your experiences using these practices.

Do you have any questions before we get started?

1. Tell me what it is like for you to work in the School District of {City} now.
2. What is it like for you to be an autism [special education] teacher in your school? Tell me about a typical day in your classroom.
3. What has it been like for you to implement these practices in your classroom?
   1. discrete trial training
   2. pivotal response training
   3. positive behavioral supports and reinforcement
   4. functional routines
   5. visual schedules and other classroom management tools

**[IF REPORT THEY DO NOT USE THESE STRATEGIES (POTENTIAL PROBES)]**

1. Tell me more about your decision to not use these practices.
   1. Whose decision was it?
2. What didn’t you like about these practices?
3. Tell me what makes it difficult to use these practices in your classroom.

**[IF REPORT THEY DON’T WANT TO OR DON’T THINK IT’S THE RIGHT PRACTICE FOR THEIR CLASS (POTENTIAL PROBES)]**

- 1. Tell me more about that.

1. What makes it easier for you to use these practices in your classroom?

**[(POTENTIAL PROBES)]**

- 1. What would you change to make these practices more relevant for you and your staff?
  2. What would you change to make these practices less burdensome for you and your staff?

1. You’ve been trained in/asked to do so many things this year. How do you make these strategies work together in your classroom?
   1. **[They don’t]: What would make it possible for you to use all of these strategies?**

**Let’s talk specifically about your school now.**

1. Tell me about your school environment. **[Clarify: what it is like as a place to work].**
   1. Tell me how the practices you use in your classroom fit within the school’s main goals and purpose.
   2. How would you describe your relationships and interactions with your support staff?
2. Who else in your school knows about what you do in your classroom?

***[(POTENTIAL PROBES)]***

- 1. How do other teachers and staff perceive your use of these practices in your classroom?
  2. Tell me how other teachers in your school support your use of these practices in your classroom.
  3. Tell me how your Special Education Liaison (SEL) supports your use of these practices in your classroom.

1. Tell me how your principal *facilitates* or *supports* your use of these practices in your classroom.
   1. How does your principal support you in learning more about these practices or other evidence-based practices for your classroom?
      1. **[They don’t]: How would you want your principal to support you?**
   2. In what ways does your principal recognize, appreciate, or reward your use of these practices with your students with autism?
      1. **[They don’t]: How would you want your principal to recognize, appreciate, or reward you?**
2. Who do you turn to in this school when you need help or have a question about autism?

***[(POTENTIAL PROBES)]***

- 1. Can you talk more about that?
  2. **[No one]: Who would you like to turn to at your school?**

1. Tell me about the most recent time your principal visited your classroom. What happened?
   1. In what ways was your principal knowledgeable about these practices that you use in your classroom?

**[(POTENTIAL PROBES)]**

- - 1. **Can you talk more about that?**
    2. **[They didn’t]: In an ideal world, what would you want your principal to do when he/she visits?**
  1. What feedback did you receive about your practices during his/her visit?

**[(POTENTIAL PROBES)]**

- - 1. **What feedback would you like to receive about your practices?**
    2. **How would you like this feedback delivered?**

1. Think of a recent challenge or barrier to using these practices in your classroom that you have experienced.
   1. What was that challenge or barrier? **[PROBE: Can you tell me more?]**
   2. Tell me the steps that your principal took to help you remove or address that barrier.
      1. **[They didn’t]: What would you have liked for them to do in that situation?**
   3. What would you want your principal to do in the future to help you address these challenges?
2. What would help you do the best job possible in your role as an autism [special education] teacher?
3. What else would you like to add that I have not asked you?

**Thank you so much for your time!**
